# Prevalence, pattern and perceptions of cleft lip and cleft palate among children born in two hospitals in Kisoro District, Uganda

**DOI:** 10.1186/1472-6831-14-104

**Published:** 2014-08-18

**Authors:** Teopista Kesande, Louis Mugambe Muwazi, Aisha Bataringaya, Charles Mugisha Rwenyonyi

**Affiliations:** 1 Department of Oral and Maxillofacial Surgery, Mulago Hospital, P.O. Box 7071, Kampala, Uganda; 2 Department of Dentistry, School of Health Sciences, College of Health Sciences, Makerere University, P.O. Box 7072, Kampala, Uganda

**Keywords:** Cleft lip, Cleft palate, Kisoro Hospital, Oro-facial, Pattern, Perceptions, Prevalence

## Abstract

**Background:**

Cleft lip with or without cleft palate is one of the most common congenital anomalies that affect the oro-facial region. The aim of the study was to determine the period prevalence, pattern and perceptions of cleft lip and cleft palate in children born between 2005 and 2010 in two hospitals in Kisoro District, Uganda.

**Methods:**

The study involved a retrospective review of medical records of mothers who delivered live babies between January 2005 and December 2010 in Kisoro Hospital and St. Francis Hospital, Mutolere in Kisoro District. Key informant interviews of mothers (n = 20) of the children with cleft lip and/or clip palate and selected medical staff (n = 24) of the two hospitals were carried out. The data were analysed using descriptive statistics.

**Results:**

Over the 6 year period, 25,985 mothers delivered live babies in Kisoro Hospital (n = 13,199) and St. Francis Hospital, Mutolere (n = 12,786) with 20 babies having oro-facial clefts. The overall period prevalence of the clefts was 0.77/1,000 live births. Sixty percent (n = 12) of children had combined cleft lip and palate and the same proportion had clefts on the left side of the face. More boys were affected than girls: 13 versus 7. About 45% of mothers were hurt on realizing that they had delivered a child with an oro-facial cleft. Forty percent of mothers indicated that a child with oro-facial cleft was regarded as an outcast. About 91.7% (n = 22) of the medical staff reported that these children were not accepted in their communities. Surgical intervention and psychosocial support were the management modalities advocated for by most respondents.

**Conclusion/recommendations:**

The period prevalence of combined cleft lip and palate in two hospitals in Kisoro District was comparable to some findings elsewhere. Cleft lip and cleft palate are a medical and psychosocial problem in Kisoro District that calls for sensitization and counseling of the families and communities of the affected children. The policy makers need to strategically plan for provision of rehabilitation with feeding obturators to facilitate easy feeding to gain weight before surgical intervention of the affected children.

## Background

Cleft lip (CL) referred to as “*harelip”*[1,2] and cleft palate (CP) are variations of a congenital deformity caused by abnormal facial development during *intra-uterine* life. The oro-facial clefts may be complete or incomplete, unilateral or bilateral, primary or secondary depending on the degree of failure of fusion of palatal shelves [1,3]. CL and CP are the most common major congenital oro-facial abnormalities and occur in approximately 1: 700 to 4:1000 live births with significant racial and geographic variation [1,3-6]. Fraser and Calnan [7] found 21% of cases had isolated CL; 33%, CP and 46% had combined CL and CP, while Melnick [8] indicated that approximately 80% of cleft lips are associated with cleft palates. Wilson [9] reported a prevalence of left sided oro-facial clefts to be twice as frequent as right sided clefts and 6-fold more frequent than bilateral clefts.

The overall incidence of associated anomalies in oro-facial cleft cases is 29%; the highest being associated with isolated cleft palate [2]. Environmental and seasonal changes are thought to influence genetic activities. Maternal tobacco smoking is reportedly associated with oro-facial clefting of the infant [10].

Children with CL or CP have associated dental anomalies, particularly the size, shape and position of the teeth [11]. Most of these children have facial deformities, speech disorders, feeding difficulties and stigma [1,12,13]. Dissatisfaction with facial appearance leads to reduced peer relationships, self esteem and intellectual competence [12-14]. Although studies on oro-facial clefts are from different populations all over the world [e.g. [1,3-6,9,10], the only available information on the Ugandan population is from one year prospective study [15] in Kampala metropolitan. Dreise et al. [15] reported a sex ratio of oro-facial clefts of 1 girl to 1.1 boys and the overall incidence of 0.73/1000 live births. The objective of the present study was to establish the period prevalence and pattern of CL and CP in children born between January 2005 and December 2010 in Kisoro District and the perception of the parents/guardians and health workers towards the children with the anomaly.

## Methods

### Study design

The study had two designs: 1) a retrospective review of mothers’ medical records and 2) interviews of key informants. The study participants were samples of convenience and were consecutively recruited.

### Study setting

The study was carried out in Kisoro Hospital and St. Francis Hospital, Mutolere in Kisoro District, Uganda. The district is hilly and is located approximately 520 km South West of Kampala. It borders with Rwanda in the south and the Democratic Republic of Congo in the west. In the east and north, it borders with Kabale and Kanungu districts, respectively, in Uganda. Kisoro District has tropical climate with 2 wet seasons from March to May and mid- September to November. The dry season from December to February means that it only rains less and remains fairly wet during these months. The second dry season from June to July is considerably drier. It is slightly warmer with daytime temperatures of about 28°C and morning temperatures of 16°C. Kisoro Hospital is the district public health facility with a 100 bed capacity and St. Francis Hospital, Mutolere is a not-for-profit catholic missionary hospital with 210 bed capacity. The two hospitals are the referral centres for the lower health units in the district. They both provide medical, surgical, obstetric/gynecologic, pediatric and other specialized services. However, there is no specialized surgical team that handles children with oro-facial clefts in the hospitals.

### Study participants

The study participants included mothers who delivered live babies with CL and/or CP between January 2005 and December 2010 in Kisoro Hospital and St. Francis Hospital, Mutolere. The medical staff members in the 2 hospitals who physically interact with mothers in the course of their work were also interviewed as key informants. The participants had to consent before recruitment into the study.

### Study variables

The dependent/outcome variables, included: prevalence, pattern and perception of cleft lip and cleft palate. The independent variables were: sex, age, tribe, place of residence (altitude), birth weight, length of gestation, birth rank, birth month (season), threatened abortion, medication during pregnancy, parental age (maternal/paternal age at the time of child birth).

### Data collection

a) Medical records

With the assistance of the Medical Records Assistants, all medical records of mothers who delivered during the period between January 2005 and December 2010 in Kisoro Hospital and St. Francis Hospital, Mutolere were retrieved from the hospital archives. The data retrieved from the medical records (Table 1) were qualitative and included demographic characteristics of the mother and the baby, as well as obstetric history, which were recorded on the data collection form. Twenty five records were excluded for being of mothers who had still births.

**Table 1:** The distribution of children according to birth weight, sex, gestation age, birth rank, threatened abortion and pattern of oral clefts (n = 20)

**Variable**	**Category**	**Number (percent)**
Hospital	Kisoro Hospital	12 (60.0)
	St. Francis Hospital, Mutolere	8 (40.0)
Birth weight of the child	< 2.5 Kg	12 (60.0)
	2.5 Kg	8 (40.0)
Sex of the child	Boy	13 (65.0)
	Girl	7 (35.0)
Birth rank of the child	1-3	5 (25.0)
	4-5	9 (45.0)
	6-7	6 (30.0)
Birth month of the child	January-March	3 (15.0)
	April-June	12 (60.0)
	July-September	4 (20.0)
	October-December	1 (5.0)
Did the mother experience threatened abortion during pregnancy?	Yes	4 (20.0)
	No	16 (80.0)
If yes, in what trimester? (n = 4)	First	2 (50.0)
	Second	2 (50.0)
	Third	0 (0.0)
Medications taken during pregnancy	Salicylates	2 (10.0)
	Penicillin	7 (35.0)
	Opiates	2 (10.0)
	Sulfanomides	3 (15.0)
	Any other	6 (30.0)
What was the maternal age?	<30 years	11 (55.0)
	≥30 years	9 (45.0)
What was the paternal age?	<30 years	5 (25.0)
	≥30 years	15 (75.0)
What type of cleft?	Cleft lip	7 (35.5)
	Cleft palate	1 (5.0)
	Cleft lip and palate	12 (60.0)
Which side of the oral cavity has the cleft?	Left	12 (60.0)
	Right	4 (20.0)
	Bilateral	4 (20.0)

b) Key informant interviews

All medical staff members who routinely and physically interacted with delivering mothers and their new born babies were requested to participate in the study. Twelve medical staff members: 1 Medical officer, 4 Clinical Officers, 5 Midwives, 1 Public Health Dental Officer and 1 Ear, Nose and Throat clinical Officer in each of the 2 hospitals were interviewed as key informants.

Based on physical addresses in the medical records, mothers of children with oro-facial clefts born between January 2005 and December 2010 were followed up and interviewed as key informants. The interviews of medical staff and mothers were carried out using pretested interview guides in English and Rufumbira language, respectively. The interviews generated both qualitative and quantitative data (Tables 2 and 3). The interview was also voice recorded verbatim to help countercheck the information that was not recorded on the form.

**Table 2:** The distribution of the mothers according to perceptions and knowledge about their children with cleft lip and or cleft palate (n = 20)

**Variable**	**Category**	**Number (percent)**
Do you have relatives who have oral clefts?	Yes	7 (35.0)
	No	13 (65.0)
What is the place of residence?	Hill	18 (90.0)
	Valley/low land	2 (10.0)
What do you think was the possible cause of the clefts?	Supernatural (evil/ancestral spirits)	11 (55.0)
	Eaten by worm	1 (5.0)
	Problems during pregnancy	2 (10.0)
	Witchcraft	1 (5.0)
	I do not know	5 (25.0)
How do people regard a child with oral cleft?	Scared	4 (20.0)
	Curiosity	2 (10.0)
	Outcast	8 (40.0)
	Normal	5 (25.0)
	I do not know	1 (5.0)
What kind of intervention do you think could be given to the child with oral cleft?	Surgical operation	12 (60.0)
	Social support/ counseling	2 (10.0)
	Surgical and social support	6 (30.0)

**Table 3:** The frequency distribution of medical staff according to knowledge and perception on cleft lip and or palate (n = 24)

**Variable**	**Category**	**Number (percent)**
What is the parents impression regarding their children’s cleft lip/palate?	Hurt	2 (8.3)
	Shocked	3 (12.5)
	Annoyed	1 (4.2)
	Disappointed	3 (12.5)
	Anxious	2 (8.3)
	Shocked and annoyed	2 (8.3)
	Disappointed and discouraged	1 (4.2)
	Disappointed and depressed	5 (20.8)
	Shocked and depressed	1 (4.2)
	No response	4 (16.7)
How do you describe the problems encountered by children with cleft lip/palate?	Feeding difficulty	5 (20.8)
	Social	3 (12.5)
	Speech	4 (16.7)
	Feeding	5 (20.8)
	Social interaction	6 (25.0)
	Infections	1 (4.2)
What is the perceived cause of oral clefts?	Drugs	4 (16.7)
	Witchcraft	2 (8.3)
	Bad omen	2 (8.3)
	Inherited	5 (20.8)
	Environmental	1 (4.2)
	I do not know	9 (37.5)
	No response	1 (4.2)
What is your assessment of community social acceptability of the cleft lip/palate?	Accepted with reservation	1 (4.2)
	Unlikely	1 (4.2)
	No	22 (91.7)
How would you describe the management modalities of a child with oral clefts?	Surgical	9 (37.5)
	Psychosocial support	1 (4.2)
	Surgical and social support	14 (58.3)
Are there any extra facial deformities in children with oral clefts?	Yes	21 (87.5)
	No	2 (8.3)
	I do not know	1 (4.2)
If yes, what are do you think are the extra-facial deformities in children with cleft lip/palate?	Webbed feet and hands	2 (8.3)
	Mental retardation	1 (4.2)
	Stunted growth	1 (4.2)
	Pierre Robin syndrome	2 (8.3)
	No response	18 (75.0)

### Data management and analysis

The filled data collection forms were double checked for errors and completeness at the end of every day’s work and the data entered into the computer using Statistical Package for Social Sciences (SPSS, version 17 for windows, Chicago, Illinois, USA). For the qualitative data, codes were derived by reading word by word and highlighting exact words from the text that appeared to capture key thoughts of the participants. Themes emerging from the codes were then summarized in frequencies and percentages and presented in Tables 2 and 3. Descriptive statistics were used to summarize the material.

### Ethical considerations

Ethical approval of the study was obtained from Makerere University Medical School Review and Ethics Committee, Kisoro District Administration, and health authorities. Written consent was obtained from the participants with maximum confidentiality in accordance with the Helsinki Declaration [16]. The participants were informed of their right to participate or opt out of the study at any stage without affecting their relationship with the investigators.

## Results

### Medical records

The occurrence of oro-facial clefts in the two hospitals was too low, hence the data were pooled. A total of 25, 985 mothers delivered live babies in Kisoro Hospital (n = 13,199) and St. Francis Hospital, Mutolere (n = 12, 786) between January 2005 and December 2010. Out of these, 20 children had oro-facial clefts: 12 in Kisoro Hospital and 8 in St. Francis, Hospital, Mutolere (Table 1). The overall period prevalence of oro-facial clefts was 0.77/1,000 births. Most children (65%, n = 13) with oro-facial clefts were boys (Table 1). Twelve (60%) of the children had combined CL and CP and with clefts on the left side of the face (Table 1). Twelve (60%) of the children had a birth weight less than 2.5 kg and gestation age of less than 37 weeks. About 45% (n = 9) of the children had a birth rank of 4th or 5th. Twelve children were born in the months of April to June. Four of the mothers of children with oro-facial clefts had a history of threatened abortion. Seven mothers were taking some penicillin based drugs during pregnancy (Table 1).

### Mothers’ interview

About 45% (n = 9) of the mothers of children with clefts reported that they were hurt on realizing that they had given birth to a child with an oral cleft. Eleven of the mothers attributed the cause of oro-facial clefts to a supernatural phenomenon (Table 2). Eight of the mothers indicated that a child with oral clefts is regarded as an outcast in their communities (Table 1). Seven of the mothers revealed that they had relatives who have oro-facial clefts. Eighteen out of twenty mothers were residing in hilly areas (Table 2). Nine children with oro-facial clefts were delivered when their mothers were more than 30 years old (Table 2). Eight mothers reported that the children with oro-facial clefts had a problem of frequent sickness.

### Medical staff interview

Twenty four medical staff members working in Kisoro Hospital (n = 12) and in St. Francis Hospital, Mutolere (n = 12) were interviewed. About 20.8% (n = 5) of the medical staff members reported that the parents get disappointed and depressed on realizing that their children had oro-facial clefts (Table 3). Five medical staff members thought that oro-facial clefts are inherited whereas nine stated that they do not know the cause (Table 3). Nine of ten medical staff members reported that a child with an oro-facial cleft is not accepted in the community. Fourteen medical staff workers stated that the management modalities of children with oro-facial clefts are surgical and psychosocial support (Table 3). Most staff members (87.5%) reported that there are other deformities in children with oro-facial clefts such as webbed feet and hands, and Pierre Robin Syndrome (Table 3). Majority of the medical staff (n = 5) indicated that the children had difficulties in feeding and recurrent infections.

## Discussion

### Methods

The present study was cross sectional in design based on medical records and key informant interviews. The interviewed mothers were consecutively selected because the cleft lip and cleft palate are rare conditions in the general population. The medical workers who routinely participate in the management of delivering mothers and new born babies were purposively recruited and interviewed as key informants as they are perceived to have interacted with mothers of children with cleft lip and palate. Thus, the findings may not represent the general population from which the respondents were drawn.

We excluded still births because they are routinely whisked away immediately for burial by relatives [15] before proper examination. Furthermore, interviewing a mother about a previous still birth could be very traumatizing. Inevitably, this exclusion could have led to loss of data. It should be noted that medical records used in the present study were for the mothers who delivered babies in the two hospitals. Although the records bore detailed physical conditions including any deformities of the baby, they lacked any identifiers. In order to avoid double documentation, records of children in the pediatric units were excluded in the study, which could have led to missing data.

We employed key informant interviews, which have the advantage of getting the information in detail. The exhaustive discussion of the questions was supplemented by translating the interview guide of the mothers into the local language (Rufumbira), which is understood by the respondents. The voice recorder was instrumental in reviewing the whole interview process and to pick some information that had not been recorded on paper during the face to face discussion.

### Findings

The overall period prevalence of oro-facial clefts in Kisoro District was 0.77/1,000 live births. This value was comparable to findings in previous study in Uganda [15] and Malawi [17] with approximately equal sample size (Table 4).

**Table 4:** The comparison of occurrence of cleft lip and cleft palate in previous and the present study

**Author**	**Population**	**Duration of study**	**Sample size**	**Proportion**
Owens et al. [18]	British	13 years	325,727	1.4/1000
Fathallah [20]	Iraqis	3 years	229,992	0.8/1000
Agbernorku et al. [5]	Ghanaians	4 years	4,000	6.3/1000
Omo-Aghoja et al. [6]	Nigerians	1 year	5,037	1.35/1000
Msamati et al. [17]	Malawians	1 year	25, 562	0.67/1000
Dreise et al. [15]	Ugandans	1 year	26,286	0.73/1000
Present study	Ugandans	6 years	25,985	0.77/1000

Cleft lip with cleft palate was the most common variation of the oro-facial clefts in the present study. This was in support of previous studies [1,7,18]. On the other hand, cleft palate alone was the least common in the two hospitals in Kisoro District (Table 1), similar to what was previously reported [1,2]. However, the previous studies had larger sample sizes and broader inclusion criteria suggestive of cautious comparison with the present study.

In the present study, the left side of the face was the most commonly affected with oro-facial clefts which is in agreement with previous findings [9]. The majority (60%) of the children with oro-facial clefts were delivered in Kisoro Hospital as compared to 40% in St. Francis Hospital, Mutolere, despite the two hospitals having approximately the same number of deliveries in the study period. The explanation for this difference is not obvious. Moreover, based on the number of deliveries in the two hospitals, the ownership of St. Francis Hospital, Mutolere (private) vesus Kisoro Hospital (public) seem to have no influence on the patient attendance.

About a third (n = 7) of the mothers admitted having relatives with cleft lip and cleft palate (Table 2), which was also echoed by some medical staff members (n = 5) who stated that oro-facial clefts are inherited conditions (Table 3) suggestive of the role of heredity [19,20]. Similar findings had previously been reported [1,3,18,21] (Table 4).

In the present study, most mothers had preterm births and infants of low birth weight (less than 2.5 kg, Table 1), which is in agreement with Pantaloni and Byrd [2]. Hagberg and co-workers [22] reported that children with oro-facial clefts and additional malformations had lower birth weight and were born earlier than children with only oro-facial clefts. Although none of any other developmental anomaly was recorded in the present study, the majority of the medical staff members (n = 21) believed there are variable frequencies of such deformities in children with oro-facial clefts (Table 3).

Pantaloni and Byrd [2] reported an association between a high incidence of oro-facial clefts and low socio-economic status, presumably due to poor nutrition in the lower end of the economic scale. However, we were not able determine the socio-economic status of the families of these children.

We noted a higher frequency of boys (n = 13) with oro-facial clefts as compared to girls (n = 7; Table 1), which is in agreement with previous studies [1,6,15,18,23].

Regarding birth rank, most children were in the 4^th^ and 5^th^ rank, implying that multi-para could be a risk factor of oro-facial clefts (Table 1). However, Pantaloni and Byrd [2] indicated that the birth order of children with oro-facial clefts is not significantly different from that of their normal counterparts.

The trend in seasonal variation of oro-facial clefts seems not to be a clear cut. In the present study most of the children were born in the months of April to June (Table 3) which corroborates a previous study [24]. Edwards [24] reported that most of the babies in Birmingham with oro-facial clefts were born in the first half of the year with a peak in the month of March. On the other hand, Pantaloni and Byrd [2] reported a higher incidence of oro-facial clefts in January and February while Owens et al. [18] indicated an association of a significant increase in the frequency of the clefts with conception in the second half of the year in Liverpool, United Kingdom.

In the present study threatened abortion was reported by 20% of the mothers. Although, Saxén [25] reported a significant association between oro-facial clefts and threatened abortion during the second trimester, he concluded that threatened abortion might be a symptom of an already malformed embryo rather than a cause of the clefting. Penicillin derivatives were the most common drugs consumed during pregnancy (Table 1). This was contrary to Niebyl [26] who indicated that penicillin derivatives are safe during pregnancy. Our findings may be coincidental bearing in mind that retrospective reporting of drug histories is prone to recall bias with no reliable evidence of compliance [18].

Most of the mothers reported that they live on the hill sides, which obviates the fact that Kisoro District is generally hilly and implies that they could have been subjected to chronic hypoxia. Castilla et al. [27] hypothesized the association of teratogenic effect of altitude on the development of the cleft lip and cleft palate, presumably due to hypertonic hypoxia.

In the present study, the majority of the mothers reported the maternal age of less than 30 years (Table 1). although we may not have established paternal age with certainty because of relying on information from third party. Pantaloni and Byrd [2] indicated that the risk of developing oro-facial clefts increases if both parents are over 30 years of age.

In support of previous findings [11], the majority of the medical staff indicated that feeding and recurrent infections were the most common problems encountered by children with oro-facial clefts (Table 3). The majority of the mothers were hurt on realizing that their children had oro-facial clefts and also thought that the cause of the clefts was a supernatural phenomenon (Table 2), which is a similar belief reported in Ghana [5]; Kenya, Russia, Cambodia, India, Egypt and Peru [28] and Nigeria [29]. Furthermore, the medical workers reported that most mothers were disappointed and depressed on realizing that they had given birth to a child with an oro-facial cleft (Table 3). Moreover, the children with cleft lip and/or cleft palate were not readily accepted in the communities and were regarded as outcasts. This finding was similarly expressed by Agbernorku and co-workers in South East Ghana [5]. The negative attitude tends to affect the child’s psychosocial development and has previously been reported [5,12-14,30,31].

In the present study, surgical intervention and psychosocial support were the recommended modalities for managing a child with cleft lip and cleft palate (Tables 2 and 3). This recommendation corroborates previous authors [28] who reported that surgical care alone is insufficient if harmful beliefs continue to victimize the affected individual. Mednick et al. [28] pointed out that care of the entire person includes providing scientific explanations and understanding of cultural beliefs that may continue to traumatize individuals with CL and/or CP even after surgical repair. However, the two hospitals in Kisoro District lacked cleft lip and cleft palate surgical teams of their own, but relied on surgical interventions under the auspices of “Flying Doctors” sponsored by African Medical and Research Foundation (St. Francis Hospital, Mutolere, Medical Superintendent, personal communication).

## Conclusions and recommendations

The period prevalence of combined cleft lip and cleft palate in Kisoro District was comparable to some findings elsewhere. Cleft lip and cleft palate are a medical and psycho-social problem in Kisoro District that calls for sensitization and counseling of the families and communities of the affected children. The policy makers need to strategically plan for provision of rehabilitation with an obturator to facilitate easy feeding to gain weight before surgical intervention of the affected children as previously recommended [32].

## Competing interests

The authors declare that there are no competing interests.

## Authors’ contributions

TK, AB, LMM and CMR contributed to the design of the study. CMR did the analysis of the data. All authors contributed equally to the preparation of the manuscript and approved the final version.

## Pre-publication history

The pre-publication history for this paper can be accessed here:

http://www.biomedcentral.com/1472-6831/14/104/prepub
